# A Modeling and Feasibility Study of a Micro-Machined Microphone Based on a Field-Effect Transistor and an Electret for a Low-Frequency Microphone

**DOI:** 10.3390/s20195554

**Published:** 2020-09-28

**Authors:** Kumjae Shin, Chayeong Kim, Min Sung, Junsoo Kim, Wonkyu Moon

**Affiliations:** 1 Safety System R&D Group, Korea Institute of Industrial Technology (KITECH), 15 Jisiksaneop-ro, Hayang-eup, Gyeongsan-si 38408, Gyeongsangbuk-do, Korea; kjshin@kitech.re.kr; 2 Department of Mechanical Engineering, Pohang University of Science and Technology (POSTECH), Pohang-si 37673, Gyeongsangbuk-do, Korea; kcycj@postech.ac.kr (C.K.); smmath2@postech.ac.kr (M.S.); chak7258@postech.ac.kr (J.K.)

**Keywords:** electret, field-effect transistor, low-frequency microphone, MEMS microphone, metal–oxide–semiconductor transistor

## Abstract

Miniaturized capacitive microphones often show sensitivity degradation in the low-frequency region due to electrical and acoustical time constants. For low-frequency sound detection, conventional systems use a microphone with a large diaphragm and a large back chamber to increase the time constant. In order to overcome this limitation, an electret gate on a field-effect transistor (ElGoFET) structure was proposed, which is the field-effect transistor (FET) mounted diaphragm faced on electret. The use of the sensing mechanism consisting of the integrated FET and electret enables the direct detection of diaphragm displacement, which leads its acoustic senor application (ElGoFET microphone) and has a strong ability to detect low-frequency sound. We studied a theoretical model and design for low-frequency operation of the ElGoFET microphone prototype. Experimental investigations pertaining to the design, fabrication, and acoustic measurement of the microphone were performed and the results were compared to our analytical predictions. The feasibility of the microphone as a low-frequency micro-electromechanical system (MEMS) microphone, without the need for a direct current bias voltage (which is of particular interest for applications requiring miniaturized components), was demonstrated by the flat-band frequency response in the low-frequency region.

## 1. Introduction

The advent of the Internet of Things has created high demand for small, high-performance micro-electromechanical system (MEMS) microphones. In particular, demand for sensors that detect low-frequency sound with high quality has increased for applications including mobile information technologies, private and military security systems, and healthcare monitoring platforms [1,2,3,4]. However, detecting low-frequency sound using commercial MEMS microphones is difficult due to the low cut-off frequency associated with the energy transduction mechanism in these microphones, namely capacitive-type energy transduction. Therefore, commercial MEMS microphones have a fundamental limitation in detecting frequencies below 100 Hz, with the reduction in capacitance and back chamber volume due to the miniaturization of the microphone.

Capacitive-type transduction measures the sensor capacitance change caused by acoustic pressure arising from the bias voltage and a load resistor, and turns the acoustic signal into an output voltage signal. As a velocity measurement of the diaphragm, the frequency response in the low-frequency region is determined by its time constant, which is the product of the transducer capacitance and load resistance [5]. Therefore, a decrease in sensor capacitance and miniaturization of a capacitive-type MEMS microphone also minimizes the change in signal charge; thus, a high-impedance load resistor or specifically designed read-out integrated circuitry is needed to measure the sound pressure wave. The ultimate result is sensor noise in the low-frequency region, and frequency response problems such as roll-off [6,7,8,9].

Acoustic backing is also a major cause of low sensitivity in the low-frequency region. Generally, the backing mechanism can be modeled as hole resistance [10] from the acoustic hole, the thin film damping effect between the diaphragm and backplate [11], and cavity compliance proportional to the volume of the back chamber [10]. Therefore, miniaturization of MEMS microphones causes roll-off problems in the low-frequency region due to acoustic resistance and compliance [12,13,14]. In other words, from both electrical and acoustic perspectives, the transduction mechanism depends upon measurement of the diaphragm velocity and leads to limitations in the low-frequency region.

To overcome this limitation, this paper describes a novel electromechanical transduction mechanism that depends on the displacement of the diaphragm, which differs from the existing velocity-based capacitive transduction. Our displacement-based transduction system introduced in this paper consists of a combination of a field-effect transistor (FET) and an electret. The electric field from the electret substrate modulates the channel of a FET embedded in the diaphragm. The FET mounted on the diaphragm vibrates due to the external acoustic pressure, which changes the distance between the channel of the FET and the electret. The conductivity of the channel is modulated by the resulting change in electric field. Thus, the use of a sensing mechanism consisting of the integrated FET and electret enables direct detection of diaphragm displacement. This transduction mechanism is produced by an electret gate on a field-effect transistor (ElGoFET). A microphone based on ElGoFET transduction is known as an ElGoFET microphone [15]. Displacement-based ElGoFET transduction enables high sensitivity and wide bandwidth in the low-frequency region. The FET is fabricated using the complementary metal–oxide–semiconductor (CMOS) process. Therefore, sensor FET and CMOS devices for signal processing can be fabricated on a single wafer, which enables monolithic fabrication of a sensor FET and a signal processing, application-specific, integrated circuit (ASIC). Because integration of the sensor and ASIC effectively reduces the electrical parasitic impedance between the sensor and signal processing circuit, it allows for miniaturization of and improvement in noise characteristics and sensitivity.

Using an analytical model, this paper analyzes theoretical sensor characteristics, particularly in the low-frequency region, of the proposed ElGoFET electromechanical transduction mechanism. Experimental investigations pertaining to the design, fabrication, and acoustic measurement of the ElGoFET microphone were performed and the results were compared to analytical predictions. Through theoretical and experimental validation, the feasibility of using the ElGoFET microphone as a low-frequency MEMS microphone, with a flat-band frequency response in the low-frequency region, was confirmed. The ElGoFET microphone, unlike conventional capacitive-type MEMS microphones, has a flat-band frequency response in the low-frequency region and does not require an additional direct current (DC) bias voltage, which is advantageous for miniaturization and low power consumption. Therefore, the ElGoFET microphone can be applied to mobile information technologies, military security systems, and healthcare monitoring systems.

## 2. Design and Operating Principle of the ElGoFET Microphone

### 2.1. Electromechanical Transduction of the ElGoFET Microphone

**Figure 1:**
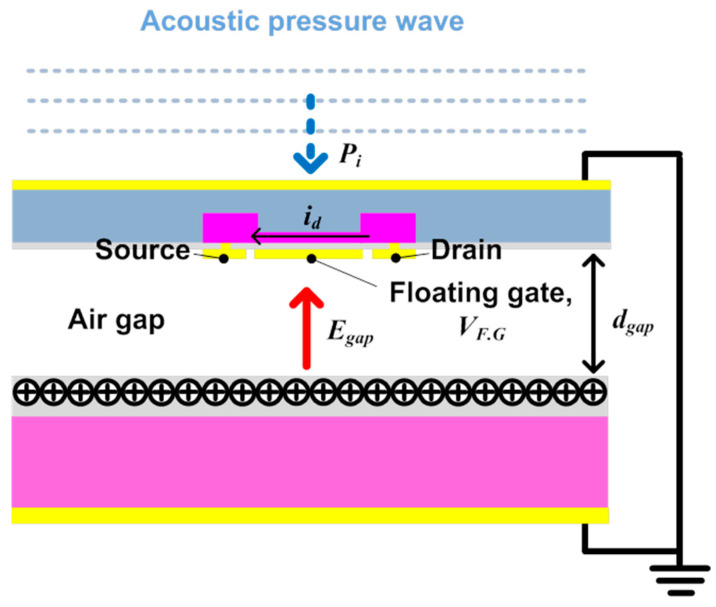
Electret gate on a field-effect transistor (ElGoFET) structure. When an external sound pressure wave is applied to the FET-embedded membrane, the gap between the FET gate and electret changes, such that the electric field applied to the FET gate fluctuates. This fluctuating electric field causes a change in the source–drain current, which represents the sound pressure wave.

**Figure 2:**
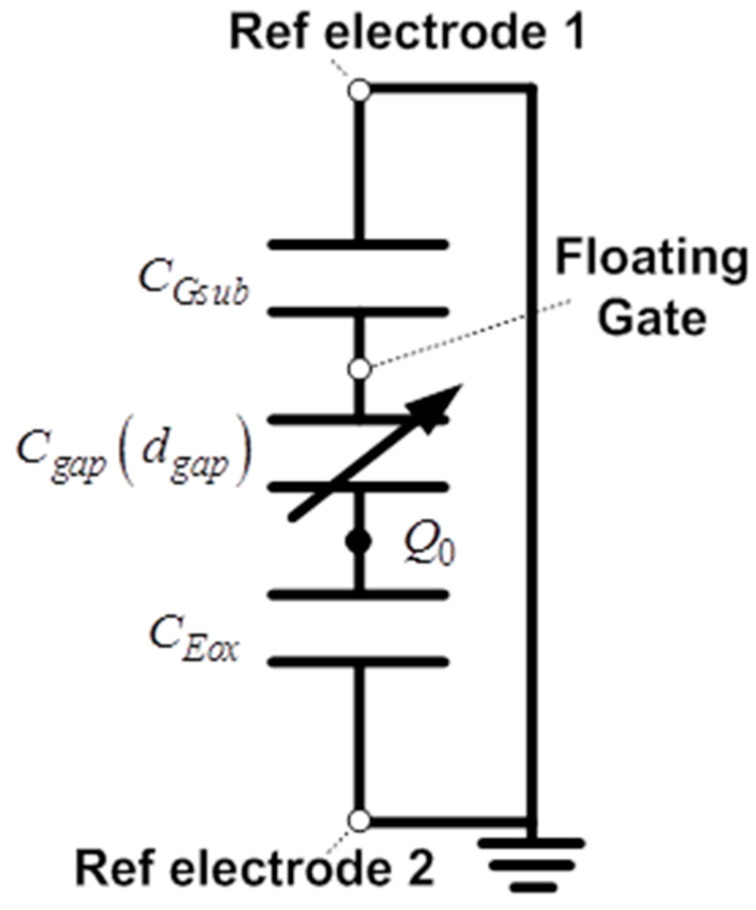
Equivalent circuit model of the ElGoFET structure. *C_Gsub_*, *C_gap_*, and *C_Eox_* represent the equivalent capacitance between the floating gate electrode and reference electrode, electret and floating gate electrode, and electret and reference electrode, respectively. The electret is expressed as a fixed charge (*Q*_0_) in the model.

Figure 1 shows the structure of the ElGoFET microphone, which consists of a dielectric layer with semi-permanently fixed charges, a diaphragm with an FET mounted in the center, and an air gap between the dielectric layer and diaphragm. A charge-trapped dielectric layer, known as the electret, is used for applying the electric field to the gate electrode. Thus, when the diaphragm vibrates in response to external sound pressure *P_i_*, the distance *d_gap_* between the diaphragm and electret changes. Because electric field intensity applied to the FET gate changes when *d_gap_* changes, the electrical potential on the floating gate electrode of the FET, *V_F.G_* also changes, which eventually affects the source−drain current *i_d_*. Figure 2 shows the electrical equivalent circuit model of the ElGoFET transduction mechanism with three capacitors, to help explain the FET gate electric potential changes induced by the electric field intensity fluctuation. *C_Gsub_*, *C_gap_*, and *C_Eox_* represent the capacitors between the floating gate electrode and the reference electrode of the FET (reference electrode 1), the electret and the floating gate electrode, and the electret and the reference electrode of the electret (reference electrode 2), respectively. Trapped charges in the electret layer are expressed in the equivalent model as a fixed charge *Q*_0_ between *C_gap_* and *C_Eox_*. Note that *C_gap_* is a function of *d_gap_*, which means that the gap capacitance is dependent on the distance between the diaphragm and floating gate electrode, as *C_gap_*(*d_gap_*). *C_Gsub_* and *C_Eox_* are constants set according to design parameters. Connecting the two reference electrodes to the ground, as shown in Figure 2, *V_F.G_* can be obtained using Equation (1), which is derived from the equivalent capacitor model considering the effects of the fixed charge *Q*_0_ [15]. This equation shows that the floating gate potential is a function of gap distance, *d_gap_*, as follows:

(1) $$V_{F.G}=\frac{C_{gap}Q_{0}}{C_{gap}C_{Gsub}+C_{Eox}C_{Gsub}+C_{Eox}C_{gap}}$$

In addition, the source−drain current *i_d_* follows the current (*I*)–voltage (*V*) square law [16], which is a general characteristic of an FET

(2) $$i_{d}=\frac{1}{2}\mu {C^{\prime }}_{ox}\frac{W}{L}{\left(V_{F.G}-V_{t}\right)}^{2}$$

where *µ* is the channel mobility, *C′_ox_* is the gate oxide capacitance per unit area, *W* and *L* are the width and length of the gate, respectively, and *V_t_* is the threshold voltage of the FET. Since *V_F.G_* is a function of *C_gap_*(*d_gap_*), as in Equation (1), and the output current signal *i_d_* is a function of *V_F.G_*, as shown in Equation (2), the ElGoFET transduction mechanism can directly measure displacement through the output current *i_d_* even under static conditions, where *d_gap_* is almost unchanged [15].

Based on this model, the electromechanical transduction sensitivity of the ElGoFET structure, *di_d_*/*dd_gap_*, was obtained. The electromechanical sensitivity of the ElGoFET transduction mechanism means that changes in the source–drain current depend on changes in the gap. Therefore, the sensitivity can be defined as the product of *dV_F.G_*_/_*dd_gap_* and *di_d_*/*dV_F.G_* by the chain rule. The floating gate potential depends on the gap variance (*dV_F.G_*/*dd_gap_*), which can be obtained by differentiating Equation (1) with respect to *d_gap,_* as follows:

(3) $$\frac{dV_{F.G}}{dd_{gap}}=\frac{-Q_{0}}{\varepsilon_{gap}A_{gap}}\frac{\frac{C_{gap}}{C_{Eox}}\frac{C_{gap}}{C_{Gsub}}}{{\left(\frac{C_{gap}}{C_{Eox}}+1+\frac{C_{gap}}{C_{Gsub}}\right)}^{2}}$$

In addition, *di_d_*/*dV_F.G_* can be derived from Equation (2), which physically denotes the transconductance of the FET, *g_m_*, as follows:

(4) $$\frac{di_{d}}{dV_{F.G}}=\mu C_{ox}^{\prime }\frac{W}{L}\left(V_{F.G}-V_{t}\right)=g_{m}$$

Therefore, the electromechanical transduction sensitivity *di_d_*/*dd_gap_* can be expressed as the product of Equations (3) and (4):

(5) $$\frac{di_{d}}{dd_{gap}}=\frac{di_{d}}{dV_{F.G}}\frac{dV_{F.G}}{dd_{gap}}=\frac{-Q_{0}}{\varepsilon_{gap}A_{gap}}\frac{\frac{C_{gap}}{C_{Eox}}\frac{C_{gap}}{C_{Gsub}}}{{\left(\frac{C_{gap}}{C_{Eox}}+1+\frac{C_{gap}}{C_{Gsub}}\right)}^{2}}g_{m}$$

From Equation (5), sensitivity is not determined only by one capacitance value, but instead by the ratio of capacitance (e.g., *C_gap_*/*C_Eox_*, *C_gap_*/*C_Gsub_*), which is a dimensionless parameter. This is an interesting aspect of the mechanism. For a conventional MEMS microphone, sensitivity is strongly dependent on the gap capacitance, and therefore on the design trade-off between the sensitivity and cut-off frequency with miniaturization. However, because the sensitivity of ElGoFET transduction is determined by the ratio of capacitance, it is possible to realize high sensitivity even with miniaturization. Although the absolute capacitance value of the sensing capacitance *C_gap_* is decreased due to miniaturization, high sensitivity can be achieved by changing the capacitance ratio. Therefore, the problems resulting from miniaturization can be resolved using ElGoFET transduction.

### 2.2. Mechanoacoustical Transduction of the ElGoFET Microphone

The electromechanical mechanism of ElGoFET transduction was described earlier. Here, we describe the acoustic sensitivity model developed for applying ElGoFET transduction to a microphone. The current sensitivity of an ElGoFET microphone can be defined as the change in source–drain current with respect to input pressure, *di_d_*/*dP_i_*. This is the product of the electromechanical sensitivity *di_d_*/*dd_gap_* and mechanoacoustical sensitivity *dd_gap_*/*dP_i_*, as expressed in Equation (6):

(6) $$\frac{di_{d}}{dP_{i}}=\frac{di_{d}}{dd_{gap}}\frac{dd_{gap}}{dP_{i}}$$

**Figure 3:**
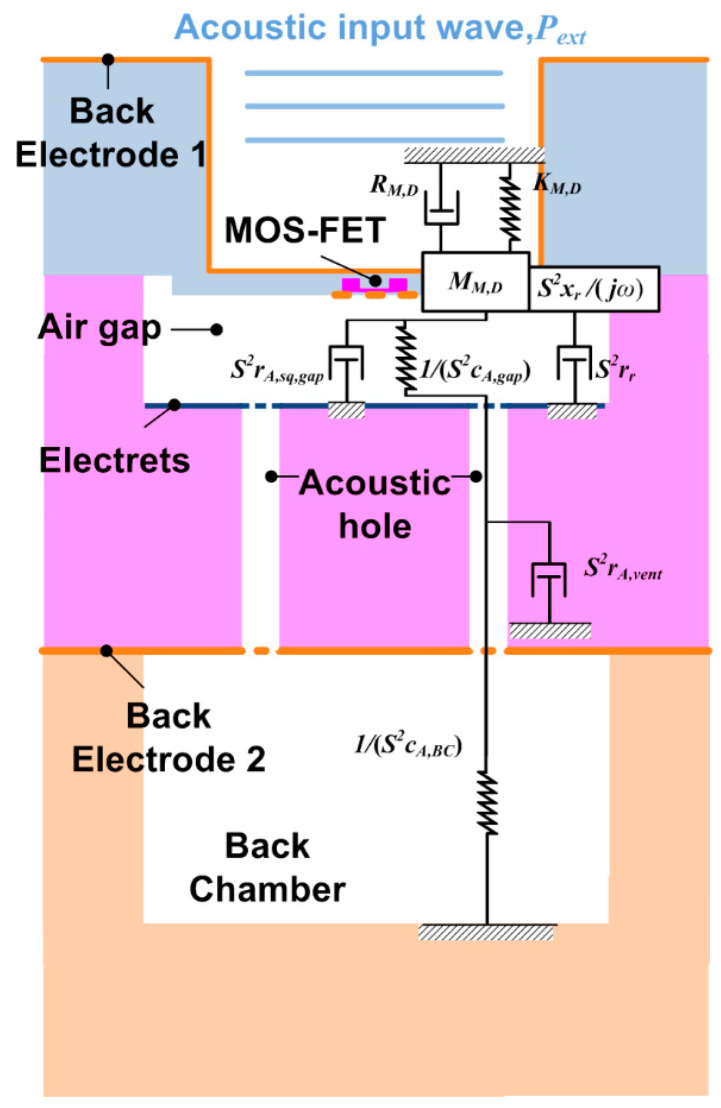
Lumped parameter model of the ElGoFET microphone. All of the mechanical and acoustical structures are expressed as lumped mass, spring, and damper components.

The mechanoacoustical sensitivity is the derivative of the displacement with respect to the input pressure, *dd_gap_*/*dP_i_*, and is determined by the diaphragm and acoustic structure of the sensor. In this study, the acoustic structure generally used in a MEMS microphone was adapted for the ElGoFET microphone, as shown in Figure 3. Specifically, to reduce the sealed impedance of the air-gap, acoustic holes were created in the electret substrate to enable fluids in the air-gap to move to the back chamber. Because MEMS microphones are much smaller than the input pressure wavelength, the ElGoFET microphone was modeled using the one-degree-of-freedom lumped element model (LEM). The LEM shown in Figure 3 incorporates two types of impedance components: mechanical (denoted with capital letters) and acoustic (represented with lowercase letters) impedance components. 

The mechanical impedance of the diaphragm is defined as

(7) $$Z_{m}=R_{M,D}+j\omega \left(\omega M_{M,D}-K_{M,D}/\omega \right)$$

where *K_M,D_*, *M_M,D_*, and *R_M,D_* are the equivalent stiffness, equivalent mass, and mechanical damping coefficient of the diaphragm, respectively, given by

(8) $$K_{M,D}=\frac{16\pi Et^{3}}{a^{2}\left(1-v^{2}\right)}$$

(9) $$M_{M,D}=\frac{192}{\Lambda_{0}}\rho_{m}\left(\pi a^{2}t\right)$$

(10) $$R_{M,D}=\frac{\sqrt{M_{M,D}K_{M,D}}}{Q_{M}}$$

where *E* is Young’s modulus, *t* is the thickness of the diaphragm, *a* is the radius of the diaphragm, *v* is Poisson’s ratio, Λ_0_ is the frequency constant, *ρ_m_* is the density of the diaphragm, and *Q_M_* is the mechanical quality factor. The acoustic radiation impedance is defined as

(11) $$Z_{r}=S^{2}\left(r_{r}+jx_{r}\right)$$

The radiation resistance *r_r_* and radiation reactance *x_r_* are defined below

(12) $$r_{r}=\frac{\rho_{0}c}{S}\left(1-\frac{2J_{1}\left(2ka\right)}{2ka}\right)$$

(13) $$x_{r}=\frac{\rho_{0}c}{S}\left(\frac{2H_{1}\left(2ka\right)}{2ka}\right)$$

where *S* is the area of the diaphragm, *ρ*_0_ is the density of the medium, *c* is the sound velocity of the medium, *J_i_*(*x*) is a first-order Bessel function, *H*_1_(*x*) is a Struve function, and *k* is the wave number. The acoustic effects of air at the back side of the diaphragm can be analyzed in a similar way using an LEM. The acoustic characteristics of the thin-film air layer between the diaphragm and electret substrate are modeled based on the compliance *c_A,gap_* caused by air compression and the squeeze film damping *r_sq_* caused by fluid viscosity. These parameters can be expressed using the following equations [17]

(14) $$c_{A,gap}=\frac{V_{gap}}{\rho_{0}c_{0}{}^{2}}$$

(15) $$r_{sq}=\frac{12\mu }{N\pi d_{gap}{}^{3}}G\left(A\right)$$

where *V_gap_* is the volume of the air gap, *µ* is the viscosity coefficient of air, and *N* is the number of acoustic holes. *G*(*A*) represent the effects of decreasing the squeeze film area due to the acoustic hole, and *A* is the area ratio with respect to the diaphragm. *G*(*A*) can be expressed using Equation (16) [11]:

(16) $$G\left(A\right)=\frac{A}{2}-\frac{A^{2}}{8}-\frac{\ln A}{4}-\frac{3}{8}$$

The air fluid resistance coefficient through an acoustic hole is given by [10,11]

(17) $$r_{A,vent}=\frac{8\mu l}{N\pi r^{4}}$$

where *l* and *r* are the length and radius, respectively, of the acoustic hole. The acoustic hole is connected to a back chamber; the representative lumped element of the back chamber is a large air volume. Considering that the back-chamber volume is much larger than the air gap volume, compliance caused by the compression of air is the only component of acoustic impedance

(18) $$c_{A,BC}=\frac{V_{BC}}{\rho_{0}c_{0}{}^{2}}$$

where *V_BC_* is the volume of the back chamber. The air gap, acoustic hole, and back chamber are connected to form an acoustic structure. The acoustic impedance of the structure on the back side of the diaphragm can be written as follows:

(19) $$Z_{b}=S^{2}\left\{r_{A,gap}+\frac{1}{j\omega c_{A,gap}}∥\left(r_{A,vent}+\frac{1}{j\omega c_{A,BC}}\right)\right\}$$

**Figure 4:**
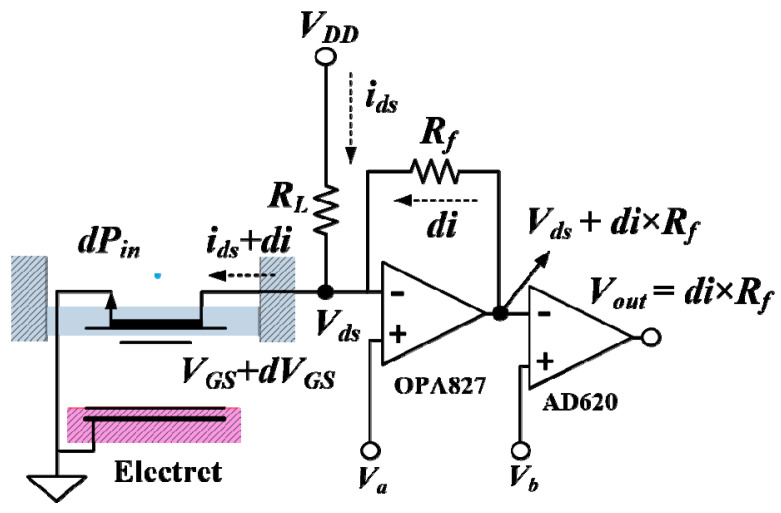
Schematic of the current–voltage (*I*–*V*) converter circuit. In the first stage, the drain supply voltage *V_DD_* and load resistor *R_L_* determine the operating point of the FET, while the feedback resistor *R_f_* represents the *I*–*V* transformation gain. In the second stage, AD620 works as an instrumentation amplifier to remove the direct current (DC) component in the output voltage.

Considering all of the impedance components of the microphone, including the diaphragm and acoustic structure, the mechanoacoustical sensitivity is given by

(20) $$\frac{dd_{gap}}{dP_{i}}=\frac{S}{j\omega Z_{total}}$$

where *Z_total_ = Z_m_ + Z_r_ + Z_b_*. Currently, the ElGoFET current sensitivity *di_d_*_/_*dP_i_* [6] can be obtained from the product of the mechanoacoustical sensitivity (Equation (20)) and the electromechanical sensitivity (Equation (5)). Figure 4 shows the *I*–*V* converter analog circuit that converts the output current signal from the ElGoFET to an output voltage signal. The feedback resistor, *R_f_*, determines the current-to-voltage transformation ratio. As such, the overall ElGoFET microphone sensitivity, which is defined as the change in output voltage signal with respect to the input sound pressure, is as follows:

(21) $$\frac{dV_{out}}{dP_{i}}=\frac{-Q_{0}}{\varepsilon_{gap}A_{gap}}\frac{\frac{C_{gap}}{C_{Eox}}\frac{C_{gap}}{C_{Gsub}}}{{\left(\frac{C_{gap}}{C_{Eox}}+1+\frac{C_{gap}}{C_{Gsub}}\right)}^{2}}\times g_{m}\times \frac{S}{j\omega Z_{total}}\times R_{f}$$

### 2.3. Design of the ElGoFET Microphone

**Figure 5:**
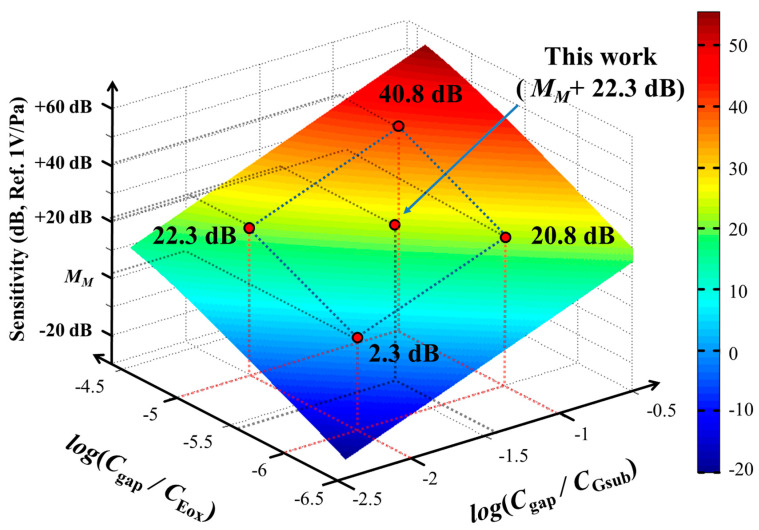
Sensitivity calculation of the ElGoFET microphone based on Equation (21).

An ElGoFET microphone was designed based on the theoretical model derived above. As represented in Equation (21), the overall sensitivity of the ElGoFET microphone was determined by the ratios of capacitance *C_gap_*/*C_Eox_* and *C_gap_*/*C_Gsub_*. Figure 5 shows the dependence of sensitivity on *C_gap_*/*C_Eox_* and *C_gap_*/*C_Gsub_*. The range of each capacitance ratio is expressed in Equations (22) and (23) as

(22) $$10^{-2}<\frac{C_{gap}}{C_{Gsub}}<10^{-1}$$

(23) $$10^{-6}<\frac{C_{gap}}{C_{Eox}}<10^{-5}$$

The reference sensitivity level *M_M_* is the mechanoacoustical sensitivity, 20log (*dd_gap_*/*dP_i_*). Therefore, Figure 5 represents the dependence of electromechanical sensitivity on the ratio of capacitance with reference to *M_M_*. The values of design parameters were determined considering both the theoretical calculation expressed in Figure 5 and the fabrication limitations of the ElGoFET microphone. First, the thickness of the gate oxide was determined to be 30 nm considering the sensitivity of the FET and its electrical stability; its length and width were 2 and 8.5 μm, respectively. The transconductance of the FET, *g_m_*, was designed to be 103 µA/V. Considering the focused ion beam (FIB) process for the electret [18], the fixed charge *Q*_0_ in the 500-nm-thick dielectric layer over a 1 × 1 mm^2^ area for the electret substrate was determined to be 7.03 nC, which was measured using an electrostatic voltmeter (Trek 323; Trek Inc., Lockport, NY, USA). The gap distance between the FET-mounted diaphragm and electret substrate, *d_gap_*, was determined to be 5 μm by considering the backing impedance effect and fabrication process. Therefore, the gap capacitance, *C_gap_*, was calculated as 1.08 fF using the commercial FEM software COMSOL Multiphysics (COMSOL, Inc., Burlington, MA, USA). The chosen capacitance ratio is expressed in Equations (24) and (25), and the sensitivity compared to the reference sensitivity level *M_M_* is 22.3 dB (1 V/Pa), as shown in Figure 5:

(24) $$\frac{C_{gap}}{C_{Gsub}}=10^{-14}$$

(25) $$\frac{C_{gap}}{C_{Eox}}=10^{-5.5}$$

**Figure 6:**
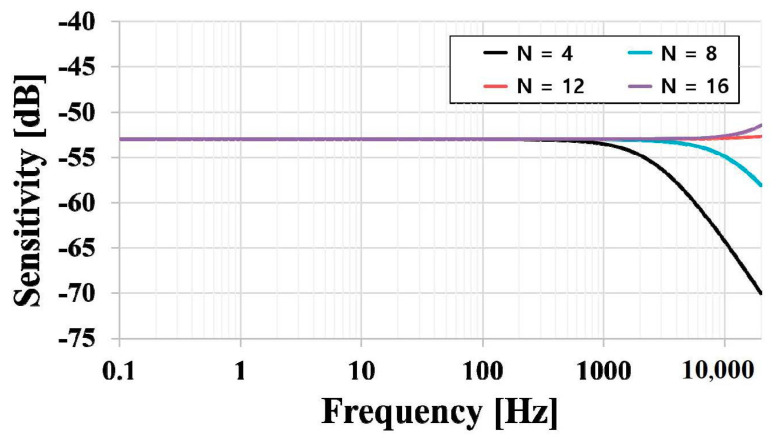
Simulation result: sensitivity–frequency response of the ElGoFET microphone.

The diaphragm of the ElGoFET microphone was designed with a diameter and thickness of 1.2 mm and 5 μm, respectively, considering stable fabrication for an FET-embedded diaphragm. The first-order resonance frequency of the designed diaphragm was 49 kHz, which ensures a flat frequency bandwidth below 20 kHz. Based on the theoretical approach and design parameters, the frequency response of the ElGoFET microphone was simulated according to the acoustic hole design parameters over a frequency ranging from 0.1 Hz to 20 kHz, as shown in Figure 6. The acoustic holes were 170 μm in diameter and 500 μm in length; the material properties used in the calculation are listed in Table 1. The frequency response changed with the number of acoustic holes. Increasing the open area by increasing the number of acoustic holes reduced the thin-film damping effect, as described in Equation (15). For this reason, the number of acoustic holes was set at 12, and the simulated sensitivity was −53 dB. In the low-frequency region, the simulated response did not exhibit the low-frequency roll-off problems typical of MEMS microphones. There was no sensitivity decrease related to the number of acoustic holes in the low-frequency region; rather, a very flat frequency response was observed.

In the capacitive transduction mechanism used in general MEMS microphones, the velocity of the diaphragm in the low-frequency region is determined by the acoustic low cut-off frequency. The acoustic holes allow the air compressed in the air gap chamber to flow out into the back chamber; consequently, the diaphragm tends to move more easily under these conditions. The acoustic hole design affects the low-frequency performance of the microphone, because the resistance of the acoustic holes and compliance of the back chamber act as an acoustic low-frequency cut-off filter. Therefore, in the conventional MEMS microphone design, acoustic holes are key to reducing the roll-off problem [6,12,13]. However, in displacement-based ElGoFET transduction, the frequency response in the low-frequency region was not strongly affected by hole resistance. Because the backing fluid resistance change related to the number of acoustic holes was negligible in terms of displacement, a flat frequency response in the low-frequency region was achieved, as shown in the simulation results. Therefore, these data provide theoretical evidence that the ElGoFET displacement sensitive transduction mechanism can effectively overcome the sensitivity degradation problem in the low-frequency region, which eventually results in a flat frequency response over a wide bandwidth, even with miniaturization.

## 3. Fabrication

The designed ElGoFET microphone was realized using micro-machining and the standard CMOS process. The diaphragm with the FET substrate and electret substrate were fabricated separately and mechanically assembled to create an ElGoFET microphone [15].

**Figure 7:**
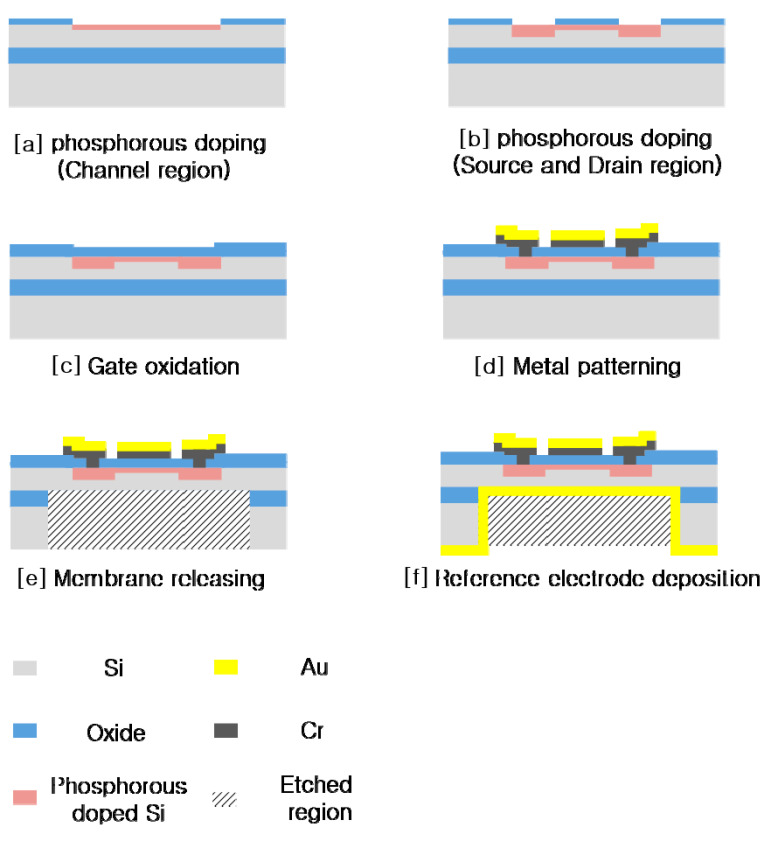
Fabrication process: FET embedded diaphragm substrate.

Figure 7 shows the fabrication process for the FET-mounted diaphragm on the FET substrate. A p-type silicon-on-insulator (SOI) wafer, with a 5-μm-thick device layer, 1-μm-thick buried oxide, and 400-μm-thick handle layer, was used for this fabrication. The channel area was defined with phosphorus doping at a dose of 2 × 10^12^ cm^−2^ using ion implantation at 20 keV (Figure 7; step [a]) for the depletion region of the depletion-mode metal–oxide–semiconductor (DMOS). The source and drain regions were doped with phosphorous at a dose of 1 × 10^15^ cm^−2^ at 80 keV (Figure 7; step [b]). The 30-nm-thick gate oxide layer was oxidized by thermal process (Figure 7; step [c]). A Cr (15 nm)/Au (150 nm) layer was used for the contact metal electrode, which was sputtered and etched by wet chemical etching after defining the contact area (Figure 7; step [d]). For the embossed pattern, inductively coupled plasma (ICP) etching was processed with 5 μm depth on the front side. To form the diaphragm structure, the back side of the diaphragm was dry-etched using deep reactive ion etching (Figure 7; step [e]). The final step of the FET-mounted diaphragm fabrication was back-side electrode deposition with Cr (15 nm)/Au (150 nm), to form the reference electrode of the FET (Figure 7; step [f]).

**Figure 8:**
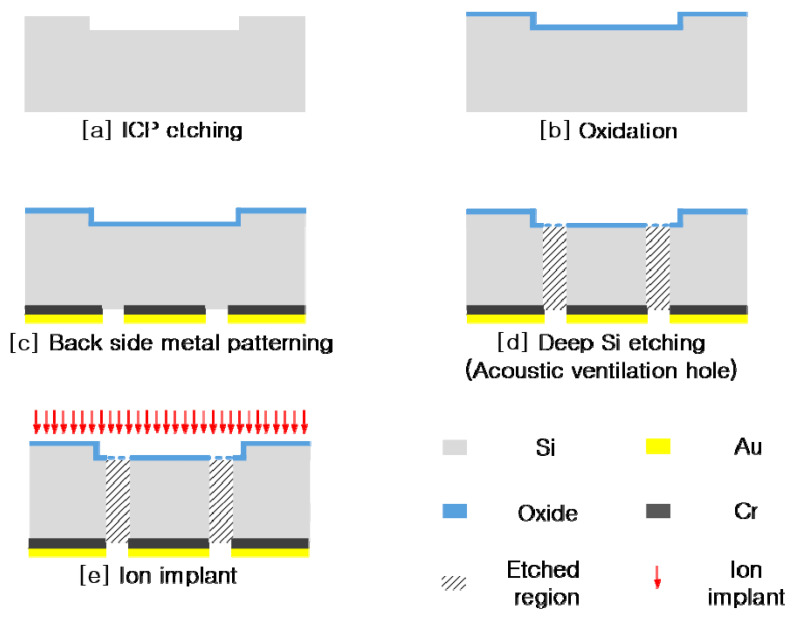
Fabrication process: electret substrate perforated by acoustic holes.

A 500-μm-thick Si wafer was used for fabricating the electret substrate, as described in Figure 8. As shown in Figure 8 (step [a]), for engraved pattern with depth of 10 μm on the front side of the bulk Si, ICP etching was processed. This engraved pattern with depth of 10 μm in the electret substrate defines the 5-μm air-gap distance of the microphone, combining with the embossed pattern in the FET substrate after the assembling process between two substrates. After the etching process, a 500-nm-thick oxidation layer was created to make the electret layer (Figure 8; step [b]), and a Cr (15 nm)/Au (150 nm) layer was patterned for reference electrode formation (Figure 8; step [c]). The acoustic holes were perforated using a dry etching process on the back of the bulk Si (Figure 8; step [d]), after which the electret substrate was released. Then, a thin hexamethyldisilazane (HMDS) layer was coated by evaporating at 140–170 °C to provide hydrophobic protection. Ga+ ion implantation was performed using an FIB at 5 keV and 0.15 nA for 1 s (Figure 8; step [e]) [18].

**Figure 9:**
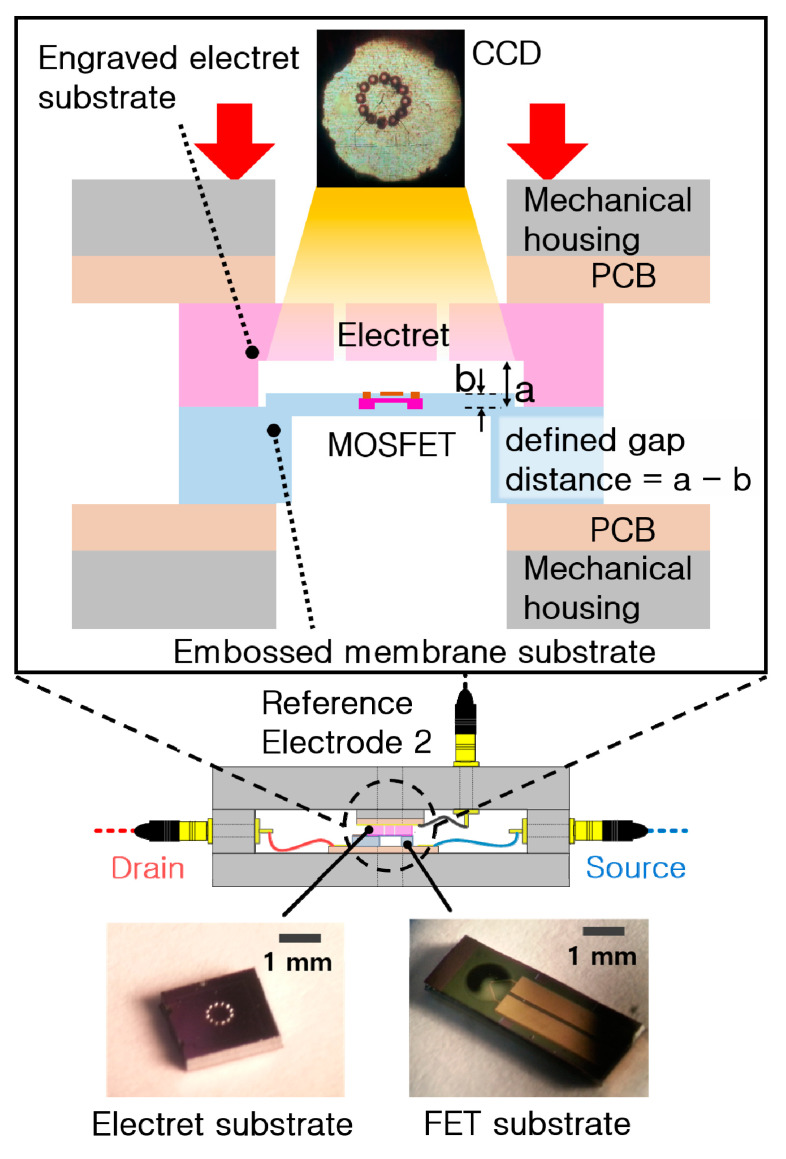
Assembly of the ElGoFET microphone: (upper) brief way to assemble; (middle) schematic diagram of an assembled ElGoFET microphone; (lower) left: electret substrate; right: FET substrate.

As a final step, the two substrates were assembled as shown in Figure 9. The FET substrate and the electret substrate on the PCBs were mounted on mechanical housing. The fabricated FET substrate and electret substrate were assembled by alignment using the mechanical manipulator. A brief way to assemble was shown in upper enlarged image in Figure 9. A delicate alignment was proceeded using a charge-coupled device (CCD). The center of FET was coincided with the center of electret surrounded with acoustic holes in assembly process. The middle image shows the assembled ElGoFET microphone’s schematic image. The lower enlarged image shows the fabricated FET substrate (left) and the electret substrate with the acoustic hole (right). While aligning and assembling the two substrates, the embossed pattern of FET substrate matched with engraved pattern of electret substrate. Then, the static gap distance between FET and electret was defined.

## 4. Experiment

### 4.1. Calibration Setup for Low-Frequency Sound Detection

**Figure 10:**
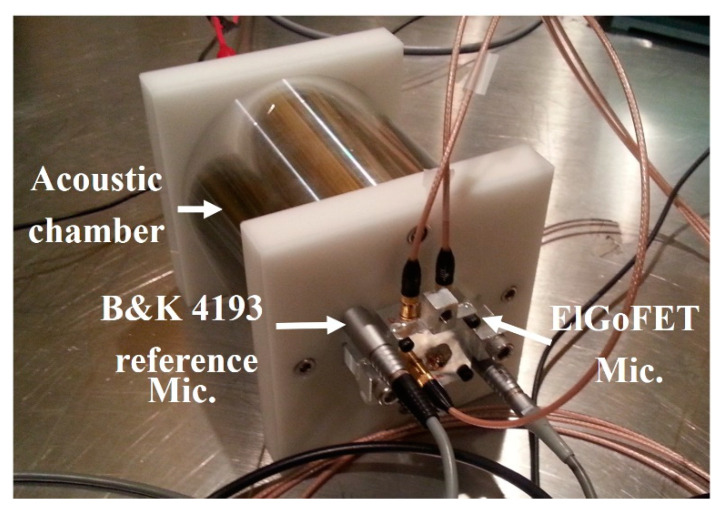
Calibration set-up for the ElGoFET microphone: small chamber for generating low-frequency acoustic calibration.

The low-frequency performance of the fabricated ElGoFET microphone was investigated. As shown in Figure 10, to generate acoustic pressure in the frequency region below 500 Hz, a small loud speaker-driven pressure chamber was designed and fabricated. The use of a closed chamber enables amplification of low-frequency acoustic pressure by the Helmholtz resonator principle, and the upper cut-off frequency can be tuned by changing the chamber volume [21,22]. Therefore, to make the chamber volume behave as an acoustic capacity element with a Helmholtz resonator, the longest dimension of the chamber must be shorter than 1/6 of the wavelength of the upper cut-off frequency of the chamber. The wavelength, λ, at 500 Hz, is 67 cm. Accordingly, the longest dimension of the chamber was designed to be 10 cm. The acoustic pressure source, a Symphony 3-inch Midrange 3–401 loudspeaker (ETON GmbH, New-Ulm, Germany) was mounted at the side of the cylinder chamber (diameter: 3 inches; length: 10 cm), and the reference microphone (B&K 4193; pressure-type, low cut-off frequency: 0.07 Hz; Bruel and Kjaer, Naerum, Denmark) and ElGoFET microphone were mounted on the other side of the chamber. The test microphones were mounted in the chamber using a tightly sealed adapter. Calibration of the ElGoFET microphone frequency response was conducted by comparison with the reference microphone. Using a frequency response analyzer (SRS 785; Stanford Research Systems, Sunnyvale, CA, USA), the frequency responses of the ElGoFET microphone and reference microphone were measured simultaneously, and their receiving characteristics were compared.

### 4.2. Experimental Results and Discussion

**Figure 11:**
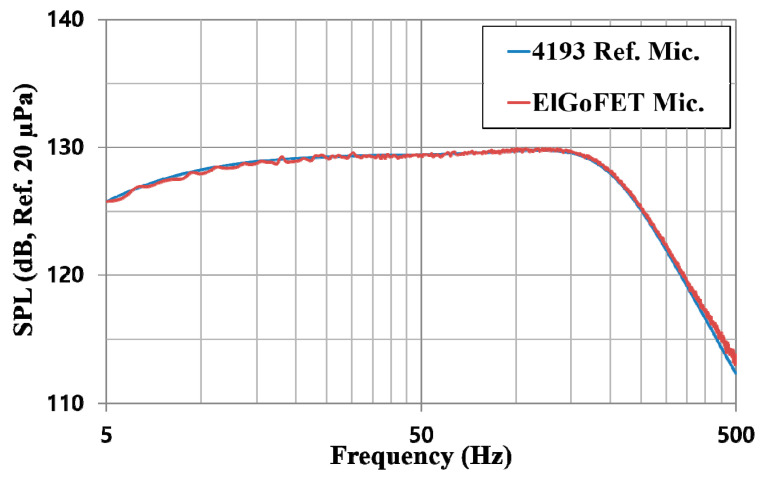
Sound pressure level (SPL) measured using the ElGoFET microphone (red line) and the B&K 4193 reference microphone (blue line).

**Figure 12:**
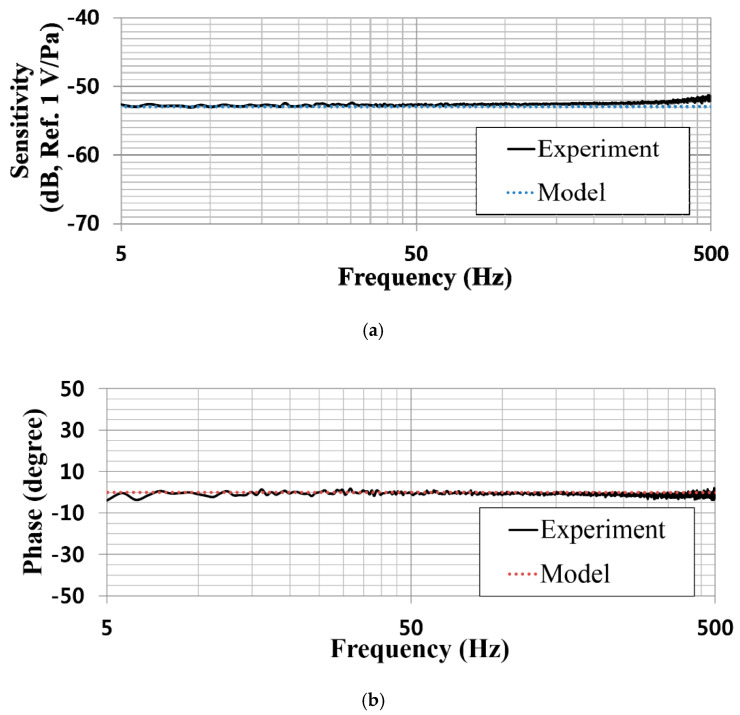
Frequency response of the ElGoFET microphone: (**a**) sensitivity and (**b**) phase.

Figure 11 shows the sound pressure levels (SPLs) measured using the reference and ElGoFET microphones over frequencies swept from 5–500 Hz; the oscillating voltage applied to the loudspeaker over the given frequency range was 500 mVpk and the feedback resistor used in the *I*–*V* converter was 680 kΩ. The SPL datasets for the two microphones were almost identical. From the measured SPL frequency response, the SPL in the chamber was as high as 126 dB at 5 Hz and about 112.5 dB at 500 Hz. The maximum SPL was about 130 dB approaching 120 Hz, which is the upper cut-off frequency of the chamber. As shown in Figure 12, the ElGoFET microphone’s sensitivity-frequency response was calibrated with reference microphone (B&K, 4193). The black solid line in Figure 12a shows the calibrated sensitivity. The sensitivity was −52.4 ± 1 dB (Ref. 1 V/Pa) in a very flat region over the 5–500 Hz band. Even in the low-frequency region around 5 Hz, sensitivity degradation due to roll-off was not observed, which proves that the displacement-based ElGoFET microphone can overcome the sensitivity degradation problem in the low-frequency region. In Figure 12a, the blue dotted line represents the theoretical prediction of the frequency response of the ElGoFET microphone based on the electro-mechano-acoustical transduction model. The theoretical prediction frequency response curve differs within about 2 dB of the experiment data (black solid line). The experimental value of the phase response also shows good agreement (within 5°) with the theoretical prediction (red dotted line), as shown in Figure 12b. The theoretical model of the ElGoFET microphone well predicted the actual performance of the fabricated ElGoFET microphone, confirming the validity of our transduction model of the ElGoFET microphone.

## 5. Conclusions and Future Work

In this study, a theoretical electro-mechanoacoustical transduction model of ElGoFET transduction for a MEMS microphone application was developed, and its validity was experimentally investigated. ElGoFET transduction is fundamentally based on displacement electromechanical transduction, because an FET-based electric field sensing mechanism is used for measuring displacement of the diaphragm. The theoretical and experimental results show excellent low-frequency response characteristics, which are not seen with conventional MEMS microphones. The measured data showed a flat sensitivity response in the low-frequency region over 5–500 Hz. This proves that the ElGoFET transduction mechanism is based on displacement-sensitive transduction, which enables the miniature MEMS microphone to have a wide frequency response in the low-frequency region. The feasibility of a low-frequency microphone using ElGoFET transduction is shown. 

The model derived for the ElGoFET microphone was developed using lumped-parameter modeling. Considering an equivalent capacitor model and the characteristic *I*–*V* equation for the FET, the electromechanical sensitivity was derived, in addition to the lumped mechanical impedance of the diaphragm, acoustic impedance of the acoustic structure, and mechanoacoustic sensitivity. By combining these elements, an integrated sensitivity model was derived and analyzed for a MEMS microphone application. Although the contribution of the electrostatic force between the electret and floating gate was not included in the electromechanical sensitivity model (because the gap capacitance was so small that it was negligible), a flat frequency response was predicted for the ElGoFET microphone by the theoretical model, and the simulation showed good agreement with measured data for both sensitivity and phase. The simulated data were in good agreement with the measured data (within ~2 dB in sensitivity and ~5° in phase). This platform provides a unified design interface based on ElGoFET transduction that could be used for other applications, such as accelerometers, hydrophones, and seismic sensors. 

For further miniaturization and performance improvement, studies of a batch wafer bonding fabrication process and optimization of the readout integrated circuit (ROIC) circuit (*I*–*V* converter), including monolithic integration with ElGoFET microphones and ASIC, are needed. The present fabrication method involves assembling the two substrates (the FET-embedded diaphragm and electret) mechanically for one ElGoFET microphone. However, this method is not applicable to batch fabrication. Thus, additional studies on batch wafer bonding processes are needed to improve miniaturization, and a refined model is needed for enhanced performance. Although the model derived in this study considered sensitivity and phase, a noise model for an ElGoFET microphone must be developed to enhance the signal-to-noise ratio. In addition, design optimization of the ROIC *I*–*V* converter and monolithic integration of an ElGoFET sensor and ASIC are required for improving noise characteristics and signal loss, and for one-chip integration of the sensor and ASIC.

## Figures and Tables

**Table 1:** Physical properties used in the simulation for the ElGoFET microphone.

Parameter	Value	Unit
Young’s modulus of silicon diaphragm, *E_Si_*	130 [19]	[GPa]
Poisson’s ratio of silicon diaphragm, *v_Si_*	0.28 [19]	-
Density of silicon diaphragm, *ρ_Si_*	2300 [19]	[kg/m3]
Frequency constant of 1st-order resonance, Λ_0_	10.22 [19]	-
Mechanical quality factor of silicon, *Q_Si_*	500 [20]	-
Viscosity of backing medium (air) *µ_air_*	18.5 [10]	[µPa·s]
Sound velocity of medium (air), *c*_0_	313 [10]	[m/s]
Density of medium (air), *ρ*_0_	1.21 [10]	[kg/m^3^]

ElGoFET: electret gate on a field-effect transistor.
